# Defining and Predicting Pain Volatility in Users of the Manage My Pain App: Analysis Using Data Mining and Machine Learning Methods

**DOI:** 10.2196/12001

**Published:** 2018-11-15

**Authors:** Quazi Abidur Rahman, Tahir Janmohamed, Meysam Pirbaglou, Hance Clarke, Paul Ritvo, Jane M Heffernan, Joel Katz

**Affiliations:** 1 Centre for Disease Modelling Department of Mathematics and Statistics York University Toronto, ON Canada; 2 ManagingLife, Inc Toronto, ON Canada; 3 School of Kinesiology & Health Science York University Toronto, ON Canada; 4 Department of Anesthesia and Pain Management Toronto General Hospital Toronto, ON Canada; 5 Department of Psychology York University Toronto, ON Canada

**Keywords:** chronic pain, pain volatility, data mining, cluster analysis, machine learning, prediction model, Manage My Pain, pain app

## Abstract

**Background:**

Measuring and predicting pain volatility (fluctuation or variability in pain scores over time) can help improve pain management. Perceptions of pain and its consequent disabling effects are often heightened under the conditions of greater uncertainty and unpredictability associated with pain volatility.

**Objective:**

This study aimed to use data mining and machine learning methods to (1) define a new measure of pain volatility and (2) predict future pain volatility levels from users of the pain management app, Manage My Pain, based on demographic, clinical, and app use features.

**Methods:**

Pain volatility was defined as the mean of absolute changes between 2 consecutive self-reported pain severity scores within the observation periods. The *k-*means clustering algorithm was applied to users’ pain volatility scores at the first and sixth month of app use to establish a threshold discriminating low from high volatility classes. Subsequently, we extracted 130 demographic, clinical, and app usage features from the first month of app use to predict these 2 volatility classes at the sixth month of app use. Prediction models were developed using 4 methods: (1) logistic regression with ridge estimators; (2) logistic regression with Least Absolute Shrinkage and Selection Operator; (3) Random Forests; and (4) Support Vector Machines. Overall prediction accuracy and accuracy for both classes were calculated to compare the performance of the prediction models. Training and testing were conducted using 5-fold cross validation. A class imbalance issue was addressed using a random subsampling of the training dataset. Users with at least five pain records in both the predictor and outcome periods (N=782 users) are included in the analysis.

**Results:**

*k-*means clustering algorithm was applied to pain volatility scores to establish a threshold of 1.6 to differentiate between low and high volatility classes. After validating the threshold using random subsamples, 2 classes were created: low volatility (n=611) and high volatility (n=171). In this class-imbalanced dataset, all 4 prediction models achieved 78.1% (611/782) to 79.0% (618/782) in overall accuracy. However, all models have a prediction accuracy of less than 18.7% (32/171) for the high volatility class. After addressing the class imbalance issue using random subsampling, results improved across all models for the high volatility class to greater than 59.6% (102/171). The prediction model based on Random Forests performs the best as it consistently achieves approximately 70% accuracy for both classes across 3 random subsamples.

**Conclusions:**

We propose a novel method for measuring pain volatility. Cluster analysis was applied to divide users into subsets of low and high volatility classes. These classes were then predicted at the sixth month of app use with an acceptable degree of accuracy using machine learning methods based on the features extracted from demographic, clinical, and app use information from the first month.

## Introduction

### Background

Digital health apps, both natively developed or Web based, are transforming how people monitor, manage, and communicate health-related information [1]. This trend has been documented in medicine [2], nursing [3], psychology [4], kinesiology [5], and nutrition [6], and multiple health concerns and diseases are being addressed [1].

Pain is one of the most prevalent health-related concerns and is among the top 3 most common reasons for seeking medical help [7]. Scientific publications of data collected from pain management apps add academic credibility to the value of digital health tools and can help both consumers and health care professionals select the right app to support their treatment plans. In a previous study [8], we applied data mining (clustering) methods to understand the engagement patterns of users from a pain management app called Manage My Pain (MMP). In that study, we divided users into 5 clusters based on their level of engagement with the app and then applied statistical methods to characterize each cluster using 6 different user attributes (eg, gender, age, number of pain conditions, number of medications, pain severity, and opioid use).

In an extension of previous work, our aim is to develop prediction models that can be used to identify and predict groups of users who report improvements or decrements in their pain experience. An important question in this effort pertains to the most appropriate statistics to use when measuring change in pain severity over time. The use of average or mean pain intensity or severity scores over time as an index of change in chronic pain has been criticized on empirical and theoretical grounds. Empirically, pain intensity among people with chronic pain tends not to change appreciably over time, given that the pain is, by definition, chronic. This is evident in treatment trials where one would expect the largest magnitude of change. For example, in a study of 1894 chronic pain patients enrolled in the Quebec Pain Registry who received state-of-the-art multidisciplinary pain treatment, a trajectory analysis showed that three-quarters of patients with moderate to severe pain intensity and pain interference scores at the start of treatment showed little to no change over a 2-year period [9]. Their pain remained relatively constant and severe (between 6/10 and 7/10) across the 24-month period. Use of average pain scores has also been criticized from a theoretical perspective in that such an approach does not account for intra- and interindividual differences over time [9,10]. To overcome these limitations, one proposed solution is to adopt different data analytical approaches such as growth mixture modeling for multivariate latent classes [9]. However, as noted above in a study that used such an approach under the ideal conditions for detecting change (ie, multidisciplinary pain treatment), the vast majority of patients did not show a change in mean pain intensity over time [9]. Similarly, within our own evaluations of the MMP database, the mean pain severity levels of most of the users did not change significantly over a 6-month period in the dataset used in this study [11]. It is important to note that the stability of mean pain scores [9,11] does not preclude the possibility that there is substantial daily intraindividual variability.

Another solution is to use a measure of change that captures fluctuation or variability in pain scores over time rather than the typical measures of central tendency (ie, mean and median) that currently dominate the pain literature. Pain volatility is an important contributor to pain experience for people with chronic pain, particularly because of its linkage with the initiation of opioid addiction [12,13]. Moreover, pain perception and consequent disability are heightened under conditions of greater uncertainty and unpredictability [14], and greater pain volatility is one of the contributors to uncertainty and unpredictability. However, as no standard definition for pain volatility exists, studies are required to evaluate the best measure of volatility and to determine the extent to which pain volatility can predict chronic pain outcomes.

### Objectives

Accordingly, this study has 2 main objectives. The first is to define a new measure of pain volatility. We apply data mining (clustering) methods on this newly defined measure to differentiate between 2 levels of volatility: high and low. The second objective is to predict users’ pain volatility level in the future based on the information extracted from their profile and the pain records created early in the *app* usage history. Logistic regression with ridge estimations, logistic regression with Least Absolute Shrinkage and Selection Operator (LASSO), Random Forests and Support Vector Machines (SVM) are employed to develop prediction models. The issue of class imbalance in the dataset is addressed through subsampling. Training and testing are conducted using standard 5-fold cross validation. Accuracy for the low and high volatility classes and overall accuracy are calculated to measure and compare the performance of prediction models developed in our experiments.

## Methods

### Manage My Pain

MMP [15], developed by ManagingLife, helps people living with pain to track their pain and functioning on a daily basis using an Android mobile phone app. As MMP was launched in 2011, >28,900 people have created an account and recorded their pain. In total, >810,000 pain episodes have been documented by users.

The central feature of MMP is the *pain record* that enables users to enter details about their pain experience. Each record contains only 1 mandatory item, a rating of pain severity using a slider on a visual analogue scale. Users have the option of completing 7 more items to more comprehensively describe their pain experience. The app issues daily reminders and prompts users to reflect on their daily accomplishments through a *daily reflection*. The completion of the daily reflection and a pain record typically takes less than 1 min to complete. With regular use, users are empowered and gain self-awareness through charts and graphs that provide insight into their pain and functioning and how it changes over time.

The information collected by the app can be summarized into a report intended for clinical use. These reports present information collected by the app in a concise fashion, primarily focusing on changes in the self-reported outcome data between clinical visits. Output is structured on a single page and tends to be more accurate than a patient’s recollection of pain since the last clinical visit, as it captures pain closer to the time of experience and is less influenced by recency and recall biases that plague existing methods for capturing pain information [9]. To supplement the information presented in the reports, users can add pain conditions, gender, age, and medications to their profile in the app. Users have the ability to use MMP without creating an account in which case data do not leave the device and are, therefore, not accessible for research such as this study.

### Procedure

This study was reviewed and approved by the research ethics board at York University (Human Participants Review Committee, Certificate #e2015-160). The users’ database was accessed and downloaded in 2 separate files (using plain text format): (1) user information and (2) pain records. The user information file contains the following fields: user ID, age at date of registration, gender, self-reported pain conditions, and self-reported medications. The pain record file contains the following fields: user ID of creator, date, severity, locations, other associated symptoms, characteristics, effective factors, ineffective factors, aggravating factors, environments, pain type, and pain duration. All fields in the text files are delimited using special characters. The files used in this study were downloaded on July 19, 2018. This study covers pain records entered by users between January 01, 2013 and July 19, 2018.

### Data

The primary dataset includes 812,548 pain records from 28,952 users. The outcome period for predicting pain volatility is the sixth month of app usage. The sixth month was chosen as the outcome period as pain lasting at least 6 months meets most generally accepted definitions of chronic pain [16]. In this study, we used the first month as the predictor period, and thus, we collected features from the first month of engagement with MMP to predict pain volatility during the sixth month of engagement with MMP. The mathematical minimum for calculating pain volatility is 2 pain records with severity ratings. However, to increase the reliability of prediction results, users with at least five pain records in both the predictor and outcome periods were considered for prediction experiments in this study. The number of users in the primary dataset that meet this criterion is 795. However, 13 users who reported *other* as their gender were excluded because of the small sample size. Thus, 782 users are selected for this study, and they have 329,070 pain records in the dataset.

### Pain Volatility

The most intuitive definition of pain volatility is the SD of pain severity ratings over time. We propose a new definition of pain volatility in this study. We define pain volatility as the mean of absolute changes between 2 consecutive pain severity ratings within each of the 2 observation periods regardless of elapsed time between pain ratings. Therefore, for a series of pain severity ratings *R=<R_1_, R_2_,…, R_n_>,* volatility, *V(R)* is defined as:

V(R) = (|R_2_-R_1_|+|R_3_-R_2_|+…+|R_n_-R_n_-1|) / n

1

**Figure 1:**
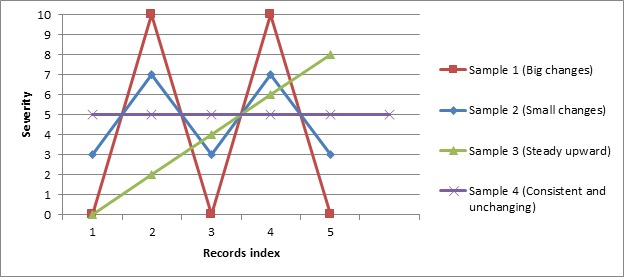
Demonstration of 4 different patterns of pain severity over time.

The differences between the mean of absolute changes as a measure of volatility and the SD measure of volatility are demonstrated in Figure 1 and Table 1 using 4 different pain scenarios. We expect the measure of volatility to demonstrate reductions (of volatility) in the following order: sample 1 (big changes), sample 2 (small changes), sample 3 (steady upward), and sample 4 (consistent and unchanging). Pain volatility defined as the mean of absolute changes conforms to this order. However, when pain volatility is defined using the SD, sample 3 (steady upward) has a higher value than that of sample 2 (small changes). From a conceptual perspective, a steady upward pattern, although conceivably distressing for a person in pain, does not conform to what we mean by pain volatility, which, by definition, involves fluctuations in pain whether from a consistent baseline (sample 1 and sample 2) or superimposed on an upward or downward trend.

Volatility can be experienced as particularly troublesome when pain severity fluctuates over time and the mean of absolute changes approximates a *saw-tooth* pattern of volatility. This pattern has previously been identified as significant in illness conditions such as atrial flutter, where hemodynamic instability can progress to ventricular fibrillation (when the heart quivers irregularly leading to an elevated risk of potentially life-threatening cardiac events) [17]. Although it remains unclear whether a saw-tooth pattern of pain volatility is more debilitating than a steady upward pattern, researchers require measurement methods elucidating both patterns to better explore associated effects on functionality and quality of life.

The next step in testing this volatility measure was to divide users into 2 distinct classes: high volatility and low volatility using a threshold on the pain volatility measure. We applied a clustering method to identify this threshold. Clustering involves partitioning a set of objects or members of a defined population into 2 or more subgroups such that the members of 1 subgroup are similar to each other but dissimilar to members of the other subgroup(s). Each object or subgroup member is represented using 1 or more variables for the purpose of clustering, which are typically referred to as features or attributes. The similarity or dissimilarity between pairs of objects (or subgroup members) is measured as the distance between the feature vectors representing them.

**Table 1:** Comparing SD of severity ratings and mean of absolute changes as pain volatility measures.

Volatility trajectory	SD of severity ratings	Mean of absolute changes
Sample 1 (Big changes)	5.48	10
Sample 2 (Small changes)	2.19	4
Sample 3 (Steady upward)	3.16	2
Sample 4 (Consistent and unchanging)	0.00	0

The output of a successful clustering process is a set of clusters where each object is assigned membership in one of the candidate clusters. We used the method known as *k-*means [18] as our primary data analytic approach to clustering users. Under the *k*-means clustering method, the number of clusters is set a priori to some constant *k*, and the dataset is partitioned into *k* clusters. In the initialization stage, the *k-*means are selected at random. Each item in the dataset is assigned to the mean closest to it. In each subsequent iteration, for each cluster, the mean is calculated based on the current members of that cluster. Each data point is then reassigned to the cluster whose mean is the closest. The iterative process stops when the cluster membership does not change between iterations.

In our experiments, the feature for clustering users is the pain volatility measure (ie, the mean of absolute changes in pain severity). We clustered the users into 2 clusters, and the volatility measure that divides the 2 groups of users was used as the threshold for defining 2 distinct classes of users: high volatility and low volatility.

### Features for Prediction Model

To develop the prediction model, we extracted the following 130 features from each of 782 users:

1. *Gender (1 feature)*: The options for entering gender in the app are male, female, or other. Users who did not include their gender information were coded as unknown. In all, 25% of users belong to this category. There were only 13 users who reported *other* as the gender. They were excluded from further analysis because of small sample size, as mentioned before in the Data subsection.
2. *Age (1 feature)*: The age (in years) recorded is the age of the user on the date of the first record and not as of the date of the analysis. We categorized the age values to facilitate the analysis and added a category to account for users with missing information. Moreover, 31% of users did not provide their date of birth. The age values are divided into 8 categories: (1) unknown, (2) >0 and ≤20, (3) >20 and ≤30, (4) >30 and ≤40, (5) >40 and ≤50, (5) >50 and ≤60, (6) >60 and ≤70, and (7) >70.
3. *Number of self-reported pain conditions (1 feature)*: Users can add 1 or more pain conditions to their profile from a centralized list of over 2500 pain conditions. They can also choose to define their own pain condition if they are unable to find one from the centralized list. Some users did not choose to add a pain condition to their profile. The number of self-reported pain conditions was divided into 5 categories: (1) unknown, (2) 1 condition, (3) 2 conditions, (4) 3 conditions, and (5) more than 3 conditions.
4. *Categories of self-reported pain conditions (5 features)*: Many of the self-reported pain conditions fit into 1 of the following 5 categories: fibromyalgia, headaches, back pain, arthritis, and depression-anxiety. Each self-reported pain condition was mapped to the appropriate category as applicable, and the mapping was reviewed for clinical correctness. For each of these 5 categories, a flag feature was created to indicate if the user has self-reported a pain condition in their profile that corresponds to the category.
5. *Pain record entries (2 features)*: A total of 2 features were used to record number of pain records in the predictor period and the number of days in the predictor period when a user has recorded at least one pain record.
6. *Pain severity rating (3 features)*: The app user must choose a pain severity rating (0-10) for each pain record created. For each user, we calculated the mean and SD of pain severity ratings from the user’s records in their predictor period. All users were also assigned to 1 of the following 3 groups based on their mean pain ratings: mild (average pain rating <4), moderate (average pain rating ≥4 to ≤7), or severe (average pain rating >7) [11]. The mean and SD of severity ratings and the severity level grouping (mild or moderate or Severe) were used as features.
7. *Change in pain trend (1 feature)*: A trend line was fitted through the pain severity ratings using linear regression. The difference in pain severity ratings between the end point and the starting point of this trend line was used as a feature.
8. *Pain volatility (2 features)*: Pain volatility in the predictor period, that is, the mean of the absolute changes between each 2 consecutive pain ratings, was used as a feature. Each user was also assigned a level of pain volatility (low or high) based on the threshold established using the clustering approach described in the previous section. This volatility level in the predictor period was used as a feature.
9. *Pain descriptors (64 features)*: For each pain record created in the app, users can report pain locations (eg, the head, abdomen, and back), associated symptoms (eg, dizziness and fever), pain characteristics (eg, burning and cramping), and environment (eg, home and school). Users can choose from a list of default values in each section: 24 pain locations, 20 associated symptoms, 13 characteristics, and 7 environments. For each of these default values, we created a flag feature indicating its presence in any of the pain records in the predictor period. Thus, there are total 64 features in this category. Only 2% of users did not report any of these pain descriptors.
10. *Factors impacting pain (43 features)*: Users in the app can report factors that may have an impact on their pain experience. A total of 3 types of factors are listed in the app: aggravating (eg, sitting and exercise), alleviating (eg, rest and sleep), and ineffective (eg, rest and sleep). Users can choose from a list of default factors in each section: 15 aggravating, 14 alleviating, and 14 ineffective. For each of these default factors, we created a flag feature indicating its presence in any of the pain records in the predictor period, resulting in 43 features in this category. In our dataset, 8% of users did not include any factor impacting their pain.
11. *Medication (5 features)*: Users can add medications to their profile from a standardized list of over 1130. Any medication in a user’s profile can be added to a pain record as an aggravating, effective, or ineffective factor. A total of 5 common categories of pain medication are identified: opioids, tricyclic antidepressants, anticonvulsants, cannabinoids, and serotonin-norepinephrine reuptake inhibitors. Medications from the standardized list are mapped to the appropriate categories. For each of these 5 categories, we created a flag feature indicating the presence of any medication that belongs to the category in any of the pain records in the predictor period. Thus, 5 features are added from the medication category.
12. *Neuropathic pain (1 feature)*: We added a flag feature as the indicator of neuropathic pain. Neuropathic pain is indicated if a user has at least two of the following in a pain record’s characteristics: pins and needles or tingling, burning, numbness, electric shocks, and light touch or clothing (aggravating factor).
13. *Mental health issues (1 feature)*: Mental health issues are indicated if a user has reported at least one of the following symptoms in a pain record: anxiety or depression (associated symptom) or negative mood or stress (aggravating factors) [19]. A flag feature was created to indicate if at least one pain record in the predictor period meets this criterion.

### Prediction Models

We first developed a logistic regression model with ridge estimators for prediction [20]. We then modified the model using LASSO [21]. These 2 logistic regression methods aim to shrink large regression coefficients to avoid overfitting. By constraining the sum of the absolute values of the coefficients, LASSO forces some coefficients to be 0 and, as such, the number of features used in the model reduces. The R package glmnet was used for training and testing logistic regression models [22,23].

We then employed 2 machine learning classifiers to build prediction models for pain volatility: Random Forests [24] and SVM [25]. Random Forests and SVM have been widely used in biomedicine for classification and prediction [26-29]. Random Forests forms an ensemble classifier based on a collection of decision trees learned from multiple random samples taken from the training set. Decision tree classifiers are constructed using the information content of each attribute; thus, the decision tree learning algorithms first select the most informative attributes for classification. Random samples from the training dataset are selected uniformly, with replacement, such that the total size of each random sample is the same as the size of the whole training set. To predict the class of a new instance, each decision tree is applied to the instance, and the final classification decision is made by taking a majority vote over all the decision trees. We applied the standard Random Forests classification package in Weka [30] using 100 trees in the Random Forests implementation. The number of features selected at random at each tree node was set to 2 √n, where n is the total number of features.

The other method, SVM, is primarily a binary linear classifier. A hyperplane is learned from the training dataset in the feature space to separate the training instances for classification. The hyperplane is constructed such that the margin, that is, the distance between the hyperplane and the data points nearest to it, is maximized. If the training instances are not linearly separable, these can be mapped into a high-dimensional space to find a suitable separating hyperplane. In our experiments, we used the Weka libsvm, employing the Gaussian radial basis function kernel.

### Measuring Prediction Performance

We used the stratified 5-fold cross-validation procedure for training the models and then testing the prediction performance. In this procedure, both low and high pain volatility users are partitioned into 5 equal-sized groups. One of these groups is used as a test set, whereas the other 4 were used to train the models and classifiers. This is repeated for each of these 5 groups. Thus, we conducted the prediction experiments 5 times, and each time, the training and test sets were completely separate. Through this cross-validation procedure, each user’s pain volatility class is tested exactly once. We measure the prediction performance of the methods used in this study by the following 3 measures:

Accuracy of the low volatility class = (Number of correctly predicted low volatility users/Total number of low volatility users) × 100%

2

Accuracy of the high volatility class = (Number of correctly predicted high volatility users/Total number of high volatility users) × 100%

3

Overall accuracy = (Number of correctly predicted low and high volatility users/Total number of users) × 100%

4

### Class Imbalance

After defining the low and high volatility classes using the clustering approach, the number of low volatility users is much higher (almost 3 times) than that of high volatility users, as discussed in the Results section. This class imbalance in the dataset produces high accuracy for the majority class (low volatility) in the prediction experiments, whereas the accuracy of the minority class (high volatility) remains very low. We used the procedure of subsampling from the majority class to create a balanced dataset for training prediction models. Under the subsampling method, instances are chosen at random from the majority class to make the size of the 2 classes equal. We repeated the subsampling procedure 3 times to ensure stability of the results. We conducted prediction experiments both on the original and the balanced dataset.

## Results

### Pain Volatility Classes

**Figure 2:**
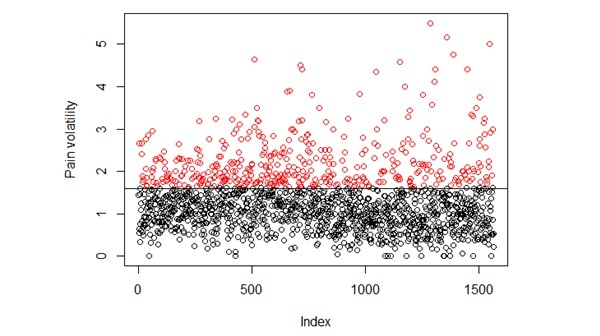
Clustering pain volatility measures. Total number of data points is 1564. Each user has 2 data points, 1 each from the predictor and outcome periods. Data points with index (x-axis) 1 to 782 are volatility values from the predictor period and 783 to 1564 are from the outcome period. Black and red colors indicate low and high volatility levels, respectively.

We combined the pain volatility measures of all users from the predictor period (ie, the first month of app usage) and the outcome period (ie, the sixth month of app usage) and then divided these data into 2 clusters using the *k*-means algorithm. Figure 2 shows the clustering output. There are a total of 1564 data points as each user has 2 values: 1 from the predictor and 1 from the outcome period. The first 782 data points (indices 1-782) are volatility values from the predictor period and the next 782 are from the outcome period. The black and red colors indicate 2 distinct classes (low and high volatility, respectively), and the numerical threshold dividing these 2 volatility classes is approximately 1.6.

**Figure 3:**
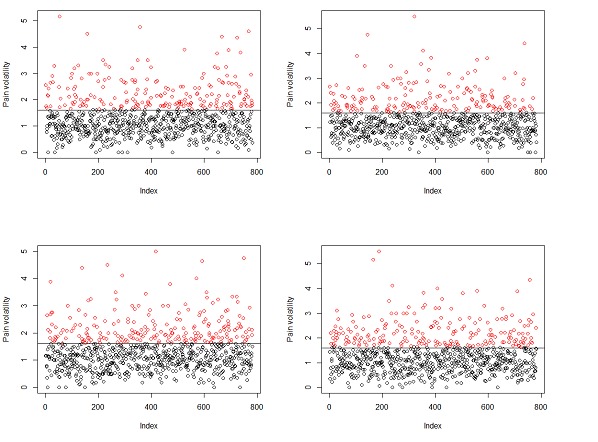
Clustering randomly selected subsets of pain volatility measures.

To further validate this threshold, we randomly chose subsamples of 782 values from the total of 1564 and reapplied the clustering algorithm. We repeated this procedure 4 times. Figure 3 shows these 4 clustering results. The threshold of 1.6 is consistent across all these 4 random subsamples. Hence, users having a volatility measure greater than 1.6 are assigned the class of *high* in our prediction experiments. All other users belong to the volatility class of *low*.

### Prediction Results

Using the pain volatility threshold of 1.6 resulted in the following division of users in the outcome period: 611 had low volatility and 171 had high volatility. There is an obvious class imbalance in the dataset as the number of low volatility users is more than 3 times the number of high volatility users. We first applied logistic regression with ridge estimators and LASSO, Random Forests, and SVM on the original dataset of 782 users. The prediction performance of 4 methods using 5-fold cross validation is presented in Table 2.

**Figure 4:**
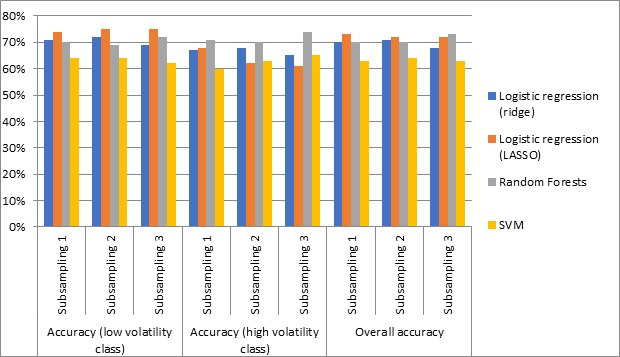
Prediction performance using the balanced dataset. LASSO: Least Absolute Shrinkage and Selection Operator; SVM: Support Vector Machines.

All 4 methods achieved 78.1% (611/782) to 79.0% (618/782) overall accuracy. However, in all methods, the accuracy of the high volatility class is significantly low. Although the accuracy for the majority class (low volatility) is more than 95.9% (586/611) across the methods, the accuracy for the minority class (high volatility) is less than 18.7(32/171). We hypothesize that the lower accuracy in the high volatility class is a result of the class imbalance. To address this, as discussed in the Methods section, we randomly subsampled the low volatility class to create training sets such that the number of instances from both classes is the same. We conducted the random subsampling 3 times and reapplied all 4 methods for prediction. The results are shown in Table 3 and Figure 4.

**Table 2:** Prediction performance using the original dataset of 782 users.

Performance measure	Logistic regression (ridge), n (%)	Logistic regression (LASSO^a^), n (%)	Random Forests, n (%)	SVM^b^, n (%)
Accuracy (low volatility class; N=611)	610 (99.8)	607 (99.3)	587 (96.1)	605 (99.0)
Accuracy (high volatility class; N=171)	1(0.6)	10 (5.3)	31 (18.1)	2 (1.2)
Overall accuracy (N=782)	611 (78.1)	617 (79.0)	618 (79.0)	607 (77.6)

^a^LASSO: Least Absolute Shrinkage and Selection Operator.

^b^SVM: Support Vector Machines.

**Table 3:** Prediction performance using the balanced dataset where random subsampling of the majority class (low volatility) was applied to make class sizes equal in the training dataset.

Performance Measure		Logistic regression (ridge), n (%)	Logistic regression (LASSO^a^), n (%)	Random Forests, n (%)	SVM^b^_,_ n (%)
**Accuracy (low volatility class; N=611)**					
	Subsampling 1	433 (70.9)	455 (74.5)	428 (70.0)	391 (64.0)
	Subsampling 2	442 (72.3)	460 (75.3)	424 (69.4)	391 (64.0)
	Subsampling 3	424 (69.4)	456 (74.6)	440 (72.0)	379 (62.0)
**Accuracy (high volatility class; N=171)**					
	Subsampling 1	115 (67.3)	116 (67.8)	121 (70.8)	103 (60.2)
	Subsampling 2	116 (67.8)	106 (62.0)	120 (70.2)	103 (63.2)
	Subsampling 3	111 (64.9)	105 (61.4)	127 (74.3)	111 (64.9)
**Overall accuracy (N=782)**					
	Subsampling 1	548 (70.1)	571 (73.0)	549 (70.2)	494 (63.2)
	Subsampling 2	558 (71.4)	566 (72.4)	544 (69.6)	499 (63.8)
	Subsampling 3	535 (68.4)	561 (71.7)	567 (72.5)	490 (62.7)

^a^LASSO: Least Absolute Shrinkage and Selection Operator.

^b^SVM: Support Vector Machines.

The overall accuracy is between 68.4% (535/782) and 73% (571/782) for Random Forests and logistic regression models. These 3 methods perform much better than SVM. Although the overall accuracy of the prediction model is reduced to some extent after balancing the dataset (Table 2 vs Table 3), the accuracy for the high volatility class is significantly improved. All methods have less than 18.7% (32/171) accuracy for the high volatility class using the original class imbalanced dataset. However, after random subsampling of the majority class, this improves to at least 60.2% (103/171). All 3 accuracy measures are approximately 70% across 3 subsamples using Random Forests. Although logistic regression models perform slightly better than Random Forests for the low volatility class, Random Forests performs better for the high volatility class. This is the only method that achieves approximately 70% accuracy consistently for both volatility classes across different subsamples. Thus, Random Forests performs the best in predicting the level of pain volatility of MMP users at the sixth month of usage based on the features collected from profile information and pain records in the first month of usage.

## Discussion

### Principal Findings

In this study, we defined a new pain volatility measure. We employed clustering methods on this measure to distinguish between low and high levels of pain volatility. Subsequently, we predicted pain volatility levels in users of MMP, a digital health app for recording pain experiences. We extracted 130 features from the users’ profile information and pain history in the first month of their app usage. These features were used to build prediction models where the outcome was the level of pain volatility in the sixth month. A total of 4 methods were used to develop prediction models: logistic regression with ridge estimators, logistic regression with LASSO, Random Forests, and SVM. We addressed the issue of class imbalance by random subsampling of the training dataset and repeated this procedure 3 times. The prediction model developed using Random Forests performs the best, and the accuracy level achieved for both low and high volatility classes is approximately 70%.

### Major Contributions

Although recent years have seen increased interest in applying machine learning methodologies in the study of chronic pain [31,32], this is the first study of its kind that aims to define and predict chronic pain volatility using data mining and machine learning methods. The results of our study are important for several reasons. First, the study involved the use of a large dataset based on real-world data from people with pain who autonomously use the app. This contrasts with the typical ways in which data are gathered by pain researchers, namely, through randomized clinical trials, surveys, and prospective trials, during which the researchers actively seek out participants. Data gathered from real-world sources have an important and complementary role to play in outcomes research and health care delivery [33]. Second, use of data from MMP, a digital health app for monitoring and tracking pain, is consistent with a recent trend in mobile health showing that similar apps are transforming how people monitor, manage, and communicate health-related information [1,2]. Third, and perhaps the most important, results from this study show that by using features of the dataset extracted over a 1-month period, we could predict pain volatility 6 months later, with a reasonably high degree of accuracy. Although the typical approach of using average pain scores may seem adequate for evaluating patterns of incrementally increasing or decreasing pain, these methods are not useful for evaluating a saw-tooth-like volatility pattern. In this study, we have explored a method that appears to reflect the quantitative levels of an important volatility pattern.

There are clinical implications to this study. Should this study on pain volatility be corroborated and shown to be a valid and reliable concept, we will be in a position to begin to identify risk factors for heightened volatility and, therefore, to potentially prevent the development of high pain volatility through effective interventions. That is, we will be able to predict patients at high risk of developing high pain volatility and the downstream negative consequences of such volatility (eg, poorer quality of life, psychosocial distress, and increased pain disability). At present, the MMP app is used for tracking and monitoring pain, and users are able to plot their pain scores as a function of time. Should pain volatility be shown to be an important, valid, and reliable construct, the app might be modified to allow users to track and plot pain volatility.

### Future Work

In future, we shall focus on selecting a subset of features that are significant predictors of pain volatility. Reducing the size of the feature set will make the prediction model easier to interpret. Furthermore, in consultation with pain experts on our team and in the broader pain community, we will validate this reduced feature set. This validated subset of features may lead to an improvement in the accuracy of prediction models as redundant features are removed. The remaining set of predictors of heightened pain volatility will be evaluated for modifiability and causality and targeted through clinical trials aimed at reducing pain volatility.

## Abbreviations

LASSO: Least Absolute Shrinkage and Selection Operator
MMP: Manage My Pain
SVM: Support Vector Machines
